# Trastuzumab Emtansine (T-DM1)-Induced Porto-Sinusoidal Vascular Disorder: A Case Report

**DOI:** 10.7759/cureus.86333

**Published:** 2025-06-19

**Authors:** Catarina L Fernandes, Orlando Pedro, Filipe Andrade, Monica Pinho

**Affiliations:** 1 Medical Oncology, Matosinhos Local Health Unit, Porto, PRT; 2 Pathology, Matosinhos Local Health Unit, Porto, PRT; 3 Internal Medicine, Matosinhos Local Health Unit, Porto, PRT

**Keywords:** breast cancer, chronic liver disease, porto-sinusoidal vascular disorder, t-dm1, trastuzumab emtansine

## Abstract

T-DM1 is used to treat early and advanced HER2-positive breast cancer and has a favorable toxicity profile. Porto-sinusoidal vascular disorder (PSVD) is a rare complication of this treatment, and its pathophysiology is not fully understood. A 43-year-old female patient was diagnosed with locally advanced HER2-overexpressing breast cancer, treated with primary chemotherapy, followed by surgery and adjuvant radiotherapy and trastuzumab. Distant relapse was identified four years later, and T-DM1 was started, leading to a complete response. Given the alterations in liver enzymes and signs of portal hypertension observed in a CT scan, a liver biopsy was performed, revealing lesions compatible with PSVD. PSVD related to T-DM1 was assumed, T-DM1 was suspended, and the patient remained under surveillance. She remained asymptomatic and with no evidence of disease for seven months. T-DM1-related PSVD is a rare complication of T-DM1 treatment that should not be overlooked. Clinical suspicion is essential for diagnosis, and T-DM1 suspension is the only known treatment.

## Introduction

Trastuzumab emtansine (T-DM1) is an antibody-drug conjugate composed of trastuzumab, a recombinant anti-human epidermal growth factor receptor 2 (HER2) monoclonal antibody, combined with emtansine (DM1), a cytotoxic drug that binds to intracellular tubulin, thereby inhibiting microtubule dynamics during cell division and leading to cell death. It is indicated for the treatment of HER2-positive recurrent or metastatic breast cancer previously treated with trastuzumab and a taxane-based chemotherapy, according to the EMILIA trial [1,2]. This trial demonstrated, besides longer progression-free survival and overall survival and a higher objective response rate, a more favorable toxicity profile when compared with the previous standard treatment (capecitabine and lapatinib).

Pharmacokinetic studies have reported that, after binding HER2, T-DM1 is internalized by hepatocytes and Kupffer cells, where it is metabolized into simpler molecules such as N-succinimidyl 4-(N-maleimidomethyl)-cyclohexane-1-carboxylate-DM1 conjugates, which are then eliminated through biliary excretion [3]. These metabolites have been shown to lead to some degree of degeneration of hepatocytes and to hyperplasia of sinusoidal cells in the liver, primarily through disruption of the hepatocytes’ microtubules, which leads to apoptosis, and through the upregulation of tumor necrosis factor alpha (TNFα), resulting in dose-dependent inflammation and necrosis of the hepatocytes [4,5]. These effects were observed with T-DM1 but not with trastuzumab or placebo, reinforcing its association with the emtansine component of T-DM1 [5]. No similar findings have been reported for other antibody-drug conjugates such as trastuzumab deruxtecan.

Notably, two frequently reported side effects of T-DM1 are elevated transaminases (16.9% to 22.4%) and thrombocytopenia (28%) [2,6-7], which may become grade 3 or higher (2.9% to 12.9%, respectively) and typically resolve with dose reduction or suspension of treatment. Since T-DM1 was introduced in clinical practice, there have been a few reports of patients who present not only with elevated transaminases and low platelet count but also with noncirrhotic portal hypertension.

Portosinusoidal vascular disorder (PSVD) is a rare and underdiagnosed condition that encompasses a group of diseases affecting portal venules and sinusoids in the absence of a defined etiology. Portal hypertension is usually associated with this condition, leading to the designation of noncirrhotic portal hypertension, but this feature is not mandatory. In asymptomatic patients, elevated transaminases, low platelet count, and/or imaging signs of portal hypertension can raise suspicion of this entity, but liver biopsy is mandatory to confirm the diagnosis [8].

PSVD can occur secondarily to several hematological, hemostatic, and immunological conditions, and it can also be induced by some drugs such as azathioprine, tioguanine, and oxaliplatin [7]. Over the last decade, T-DM1 has also been identified as a potential contributor to this disorder. The real incidence of PSVD associated with T-DM1 is unknown, but there are currently fewer than 20 cases described in the literature [9-15]. Most of the reported cases involve patients who were heavily pretreated, with a median of 4 (2-8) previous lines of cytotoxic therapy, and PSVD was documented to occur 11 to 30 months after T-DM1 initiation. None of the patients had evidence of secondary liver lesions.

## Case presentation

We report the case of a 43-year-old woman with no relevant previous medical or family history who was diagnosed with grade 3 invasive breast carcinoma with overexpression of HER2 (score 3+ by immunohistochemistry) and negative hormonal receptors, initially staged as cT2N+ cM0, seven years ago. The patient was initially treated with neoadjuvant doxorubicin 600 mg/m² and cyclophosphamide 60 mg/m² (AC) every three weeks for four cycles, followed by four cycles of docetaxel 75 mg/m² and trastuzumab 600 mg administered subcutaneously every three weeks, after which she underwent lumpectomy. The histopathological exam revealed stage ypTisN0. The patient was then treated with adjuvant radiotherapy and trastuzumab 600 mg, administered subcutaneously every three weeks until she completed a full year of treatment.

**Figure 1:**
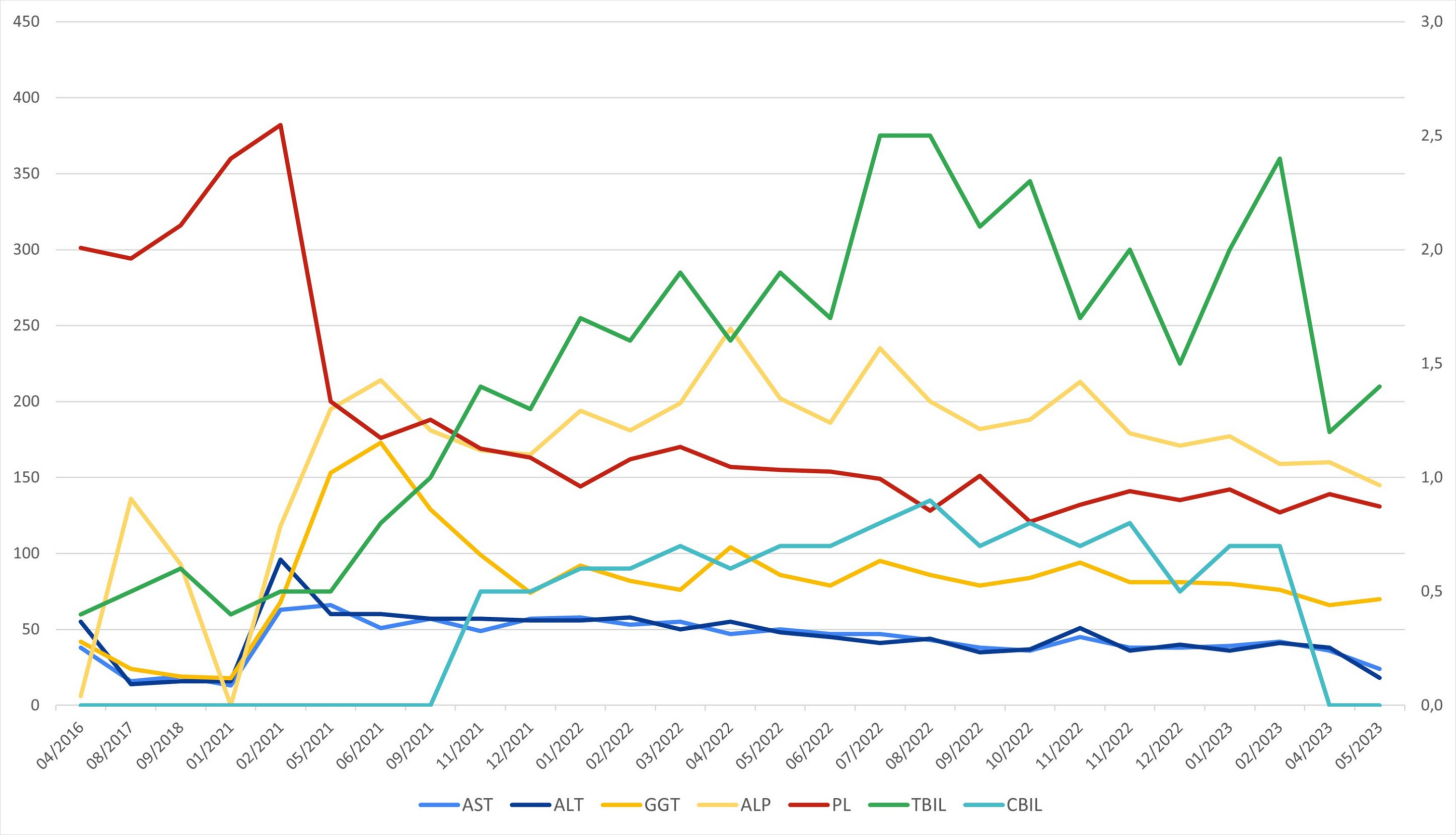
Graph showing the evolution of platelet count (×10³ per microliter), bilirubin (mg/dL), and liver enzymes (U/L) throughout treatment with T-DM1 (created by the authors). AST: aspartate aminotransferase, ALT: alanine aminotransferase, GGT: gamma-glutamyl transferase, ALP: alkaline phosphatase, PL: platelets, TBIL: total bilirubin, CBIL: conjugated bilirubin.

After three and a half years of surveillance, disease recurrence was identified, with an ipsilateral major pectoralis muscle lesion associated with lung and broncho-hilar nodal metastases. The major pectoralis muscle lesion was biopsied, and histological examination confirmed a breast carcinoma metastasis, maintaining HER2 overexpression (immunohistochemistry staining score 3+) and negative hormonal receptors. No liver metastases were identified. After the diagnosis of metastatic disease, the patient maintained a good performance status, with an Eastern Cooperative Oncology Group performance status (ECOG PS) of 0, and began first-line palliative chemotherapy with T-DM1 at 3.6 mg/kg. From the first cycle of T-DM1, the patient experienced a very slight but persistent grade 1 elevation of transaminases and gamma-glutamyl transferase, which were previously normal (see Figure 1). After nine months of treatment, the patient also developed grade 1 elevation of bilirubin and grade 1 thrombocytopenia. A review of the patient’s medications revealed no new drugs or supplements with potential hepatotoxic effects. Hyperbilirubinemia gradually increased until it became grade 2, approximately 15 months after starting T-DM1. In response, the T-DM1 dose was reduced to 3 mg/kg (level 1 reduction), and the patient continued treatment. However, after two months on the reduced dose, grade 2 hyperbilirubinemia recurred, prompting a second dose reduction to 2.4 mg/kg. The patient remained asymptomatic, with a normal coagulation study (INR 1.2) and no hypoalbuminemia, but she consistently exhibited grade 1 elevation of transaminases. Figure 1 and Table 1 summarize the evolution of the patient's liver enzymes, bilirubin, and platelet count during treatment with T-DM1.

**Table 1:** Evolution of liver enzymes, bilirubin, and platelet count throughout treatment with T-DM1. AST: aspartate aminotransferase, ALT: alanine aminotransferase, GGT: gamma-glutamyl transferase, ALP: alkaline phosphatase.

Date	AST, U/L (Reference: 5-34)	ALT, U/L (Reference: <55)	GGT, U/L (Reference: <55)	ALP, U/L (Reference: 40-150)	Total bilirubin, mg/dL (Reference: 0.2-1.2)	Conjugated bilirubin, mg/dL	Platelet count, x10^3^/mcL (Reference: 150-400)
04/2016	38	55	42	<6	0.4		301
08/2017	16	14	24	136	0.5		294
09/2018	19	16	19	93	0.6		316
01/2021	13		18		0.4		360
02/2021 (T-DM1 started)	63	96	68	118	0.5		382
19/05/2021	66		153	195	0.5		200
30/06/2021	51	60	173	214	0.8		176
01/09/2021	57	57	129	181	1		188
03/11/2021	50	40	115	192	1.3	0.4	165
24/11/2021	49		99	168	1.4	0.5	169
05/01/2022	57	56	74	165	1.3	0.5	163
26/01/2022	58		92	194	1.7	0.6	144
16/02/2022	53	58	82	181	1.6	0.6	162
09/03/2022	43	41	68	203	1.6	0.7	165
30/03/2022	55	50	76	199	1.9	0.7	170
07/04/2022	47	49	79	201	1.7	0.7	166
27/04/2022	47	55	104	248	1.6	0.6	157
18/05/2022	50	48	86	202	1.9	0.7	155
26/05/2022	58	54	92	215	1.6	0.7	141
15/06/2022 (1st dose reduction)	47	45	79	186	1.7	0.7	154
07/07/2022	47	41	95	235	2.5	0.8	149
15/07/2022	42	38		188	2	0.8	131
21/07/2022	41	38	87	190	1.7	0.7	138
11/08/2022 (2nd dose reduction)	43	44	86	200	2.5	0.9	128
08/09/2022	49	48	94	228	1.8	0.7	133
28/09/2022	38	35	79	182	2.1	0.7	151
12/10/2022	36	37	84	188	2.3	0.8	121
03/11/2022	45	51	94	213	1.7	0.7	132
17/11/2022	38	34	82	192	2.3	0.8	104
23/11/2022	38	36	81	179	2	0.8	141
02/12/2022	35	34		179	2	0.6	133
15/12/2022	34	33	79	174	2.3	1	136
28/12/2022 (T-DM1 suspension)	38	40		171	1.5	0.5	135
19/01/2023	39	36		177	2	0.7	142
13/02/2023	42	41	76	159	2.4	0.7	127
04/04/2023	36	38	66	160	1.2		139
24/05/2023	24	18					131
12/07/2023	36	97	70	145	1.4	0.5	71

**Figure 2:**
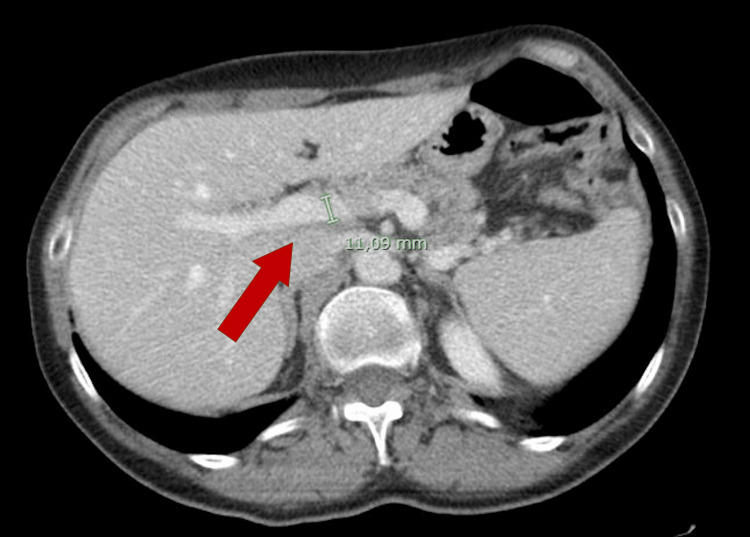
CT scan before T-DM1 treatment was started, showing normal portal vein diameter (red arrow).

**Figure 3:**
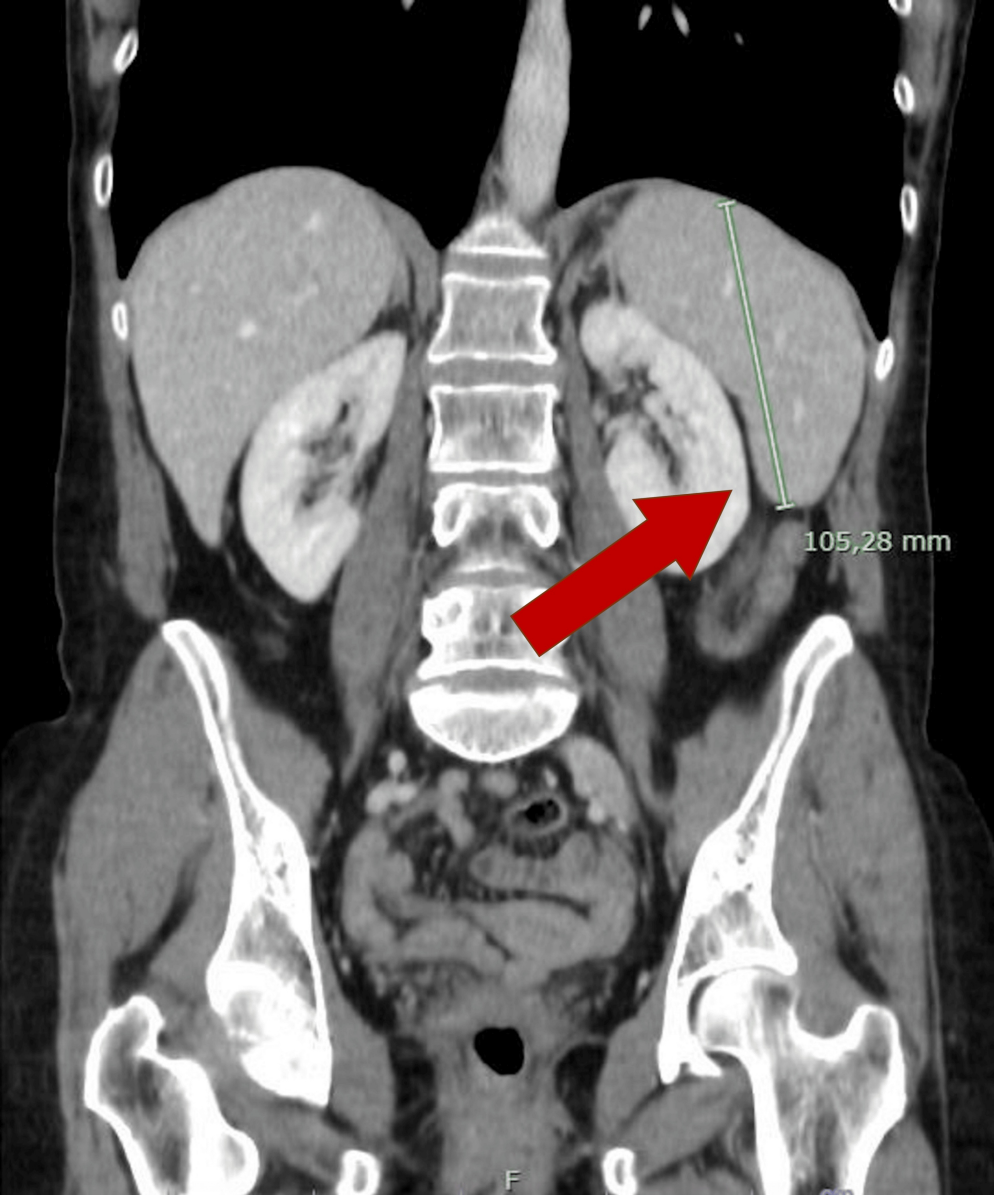
CT scan before T-DM1 treatment was started, showing normal spleen size (red arrow).

**Figure 4:**
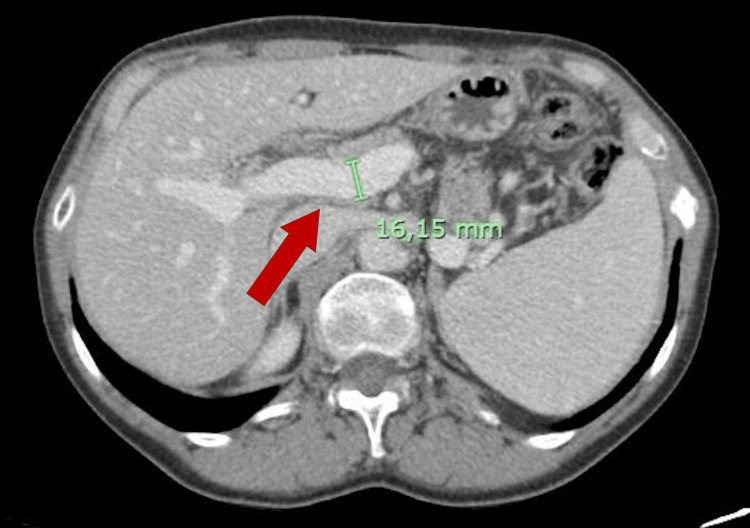
CT scan 19 months after T-DM1 treatment was started, showing de novo enlarged portal vein (red arrow).

**Figure 5:**
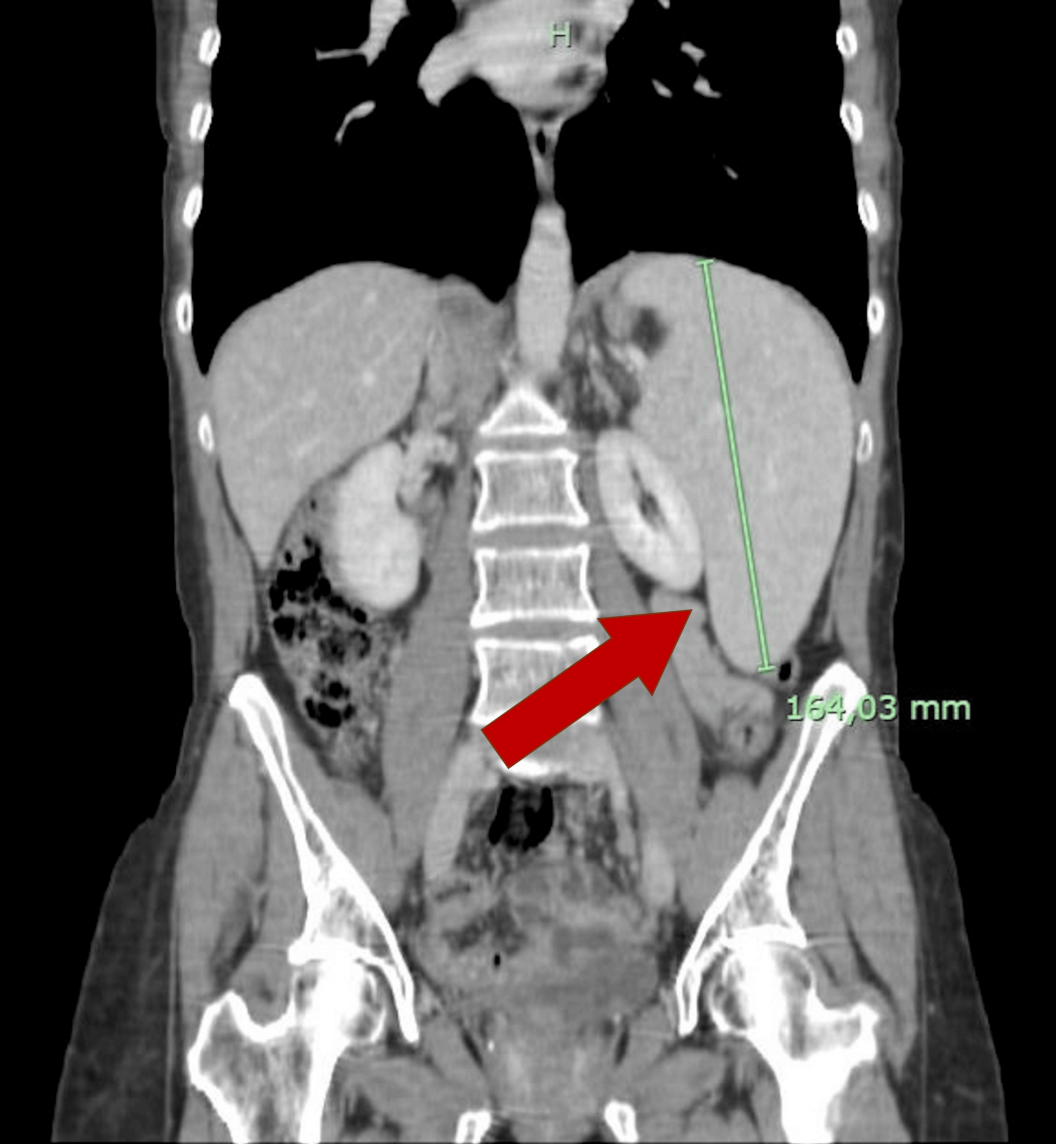
CT scan 19 months after T-DM1 treatment was started, showing de novo splenomegaly (red arrow).

**Figure 6:**
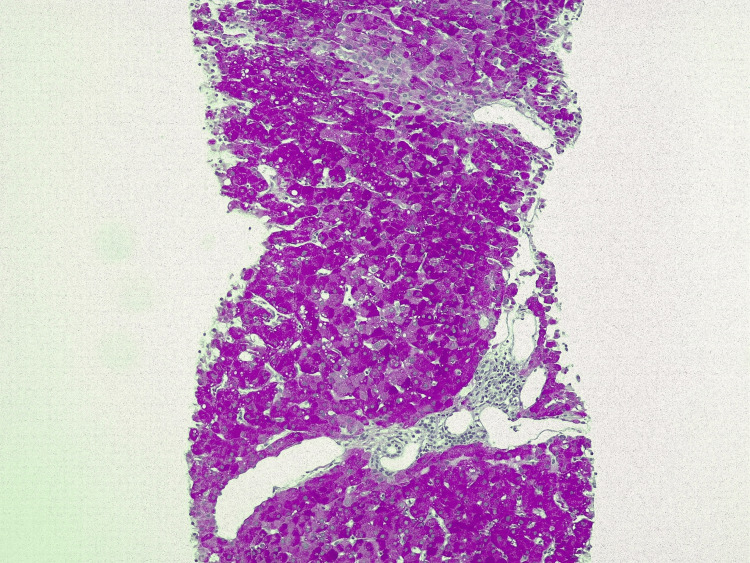
Liver biopsy with periodic acid-Schiff (PAS) staining showing liver parenchyma with preserved trabecular architecture, dilated vessels in the periphery of portal spaces, and lobular parenchyma with mild sinusoidal enlargement (10x magnification).

**Figure 7:**
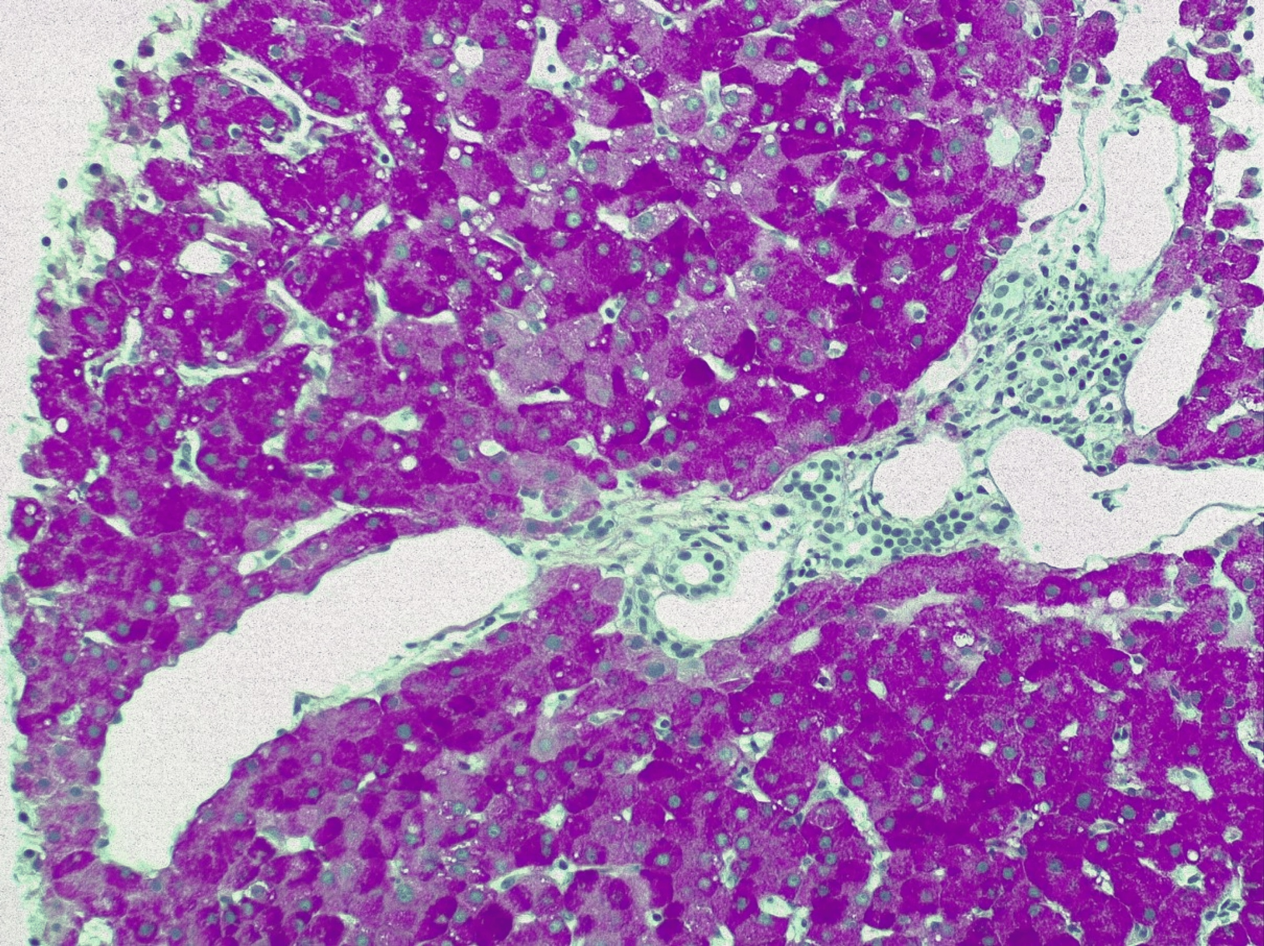
Liver biopsy with periodic acid-Schiff (PAS) staining showing liver parenchyma with preserved trabecular architecture, dilated vessels in the periphery of portal spaces, and lobular parenchyma with mild sinusoidal enlargement (20x magnification).

A surveillance computed tomography (CT) scan performed 18 months after initiating T-DM1 treatment revealed portal vein enlargement (16.2 mm) and newly detected splenomegaly (142 mm) (Figures 2-5), raising suspicion of portal hypertension. A full liver disease study was performed, revealing negative viral hepatitis serologies, negative hepatic autoantibodies (e.g., ANA, AMA, LKM-1, ASMA), no evidence of iron overload, normal electrophoresis results and immunoglobulin count, and normal levels of alpha-1 antitrypsin, ceruloplasmin, and alpha-fetoprotein. No liver metastases were identified on imaging studies. Liver elastography results were normal, but the hepatic venous pressure gradient was elevated (11 mmHg), confirming sinusoidal portal hypertension. Liver biopsy findings showed an absence of fibrosis, dilated vessels around portal spaces, and sinusoidal dilatation (Figures 6, 7). These findings, together with the timing of the onset of liver enzyme abnormalities, strongly indicated a diagnosis of PSVD related to T-DM1.

During the first six months of treatment with T-DM1, thoracoabdominopelvic CT scan demonstrated complete radiologic response. After the etiologic investigation was concluded and the diagnosis confirmed, T-DM1 was suspended following 22 months of treatment. The patient was initiated on low-dose spironolactone (25 mg daily) and furosemide (20 mg daily). The multidisciplinary board recommended keeping the patient under active surveillance, considering that she remained with no evidence of disease.

Liver enzyme levels and hyperbilirubinemia normalized within three months after T-DM1 discontinuation, but grade 1-2 thrombocytopenia persisted even after five months. An abdominal magnetic resonance imaging (MRI) performed seven months after T-DM1 suspension revealed further enlargement of the portal vein (21 mm) and the presence of esophageal varices. An upper endoscopy attempt was unsuccessful because the patient could not tolerate the procedure without sedation, and she is currently awaiting rescheduling. The patient remains asymptomatic, with no evidence of ascites or encephalopathy, and is currently seven months into surveillance with no signs of recurrent disease.

## Discussion

T-DM1 is a very useful treatment option for HER2-positive metastatic and early breast cancer, offering excellent outcomes in terms of disease-free, progression-free, and overall survival, along with a generally favorable toxicity profile. Nevertheless, adverse reactions are common and occasionally lead to dose reduction or discontinuation of the drug. Hepatotoxicity and hematological toxicity are expected, given T-DM1 metabolization, excretion, and internalization into megakaryocytes. As a result, elevated transaminases, thrombocytopenia, and hyperbilirubinemia are all relatively common adverse events, though they usually resolve with dose reduction and are rarely related to vascular liver toxicity.

This report describes a rare confirmed case of T-DM1-related PSVD, adding to the nearly 20 cases currently described in the literature. In this case, elevation of transaminases occurred shortly after treatment initiation; however, it was mild and remained stable throughout the treatment. Persistent grade 2 hyperbilirubinemia served as an alarm sign, prompting further investigation and ultimately leading to a diagnosis of PSVD. A high level of suspicion was essential for diagnosis, especially in an asymptomatic patient. Besides the typical histological findings, the close temporal association with T-DM1 treatment was critical for the diagnosis.

The time to PSVD diagnosis in this patient (22 months) exceeded the reported median of fewer than 18 months. Even so, it was timely enough to prevent severe liver disease and further complications. Unlike most described cases, this patient had not undergone extensive prior chemotherapy, highlighting the isolated role of T-DM1 in the pathogenesis of PSVD. Clinical suspicion leading to appropriate investigation and early diagnosis is of great importance, as T-DM1 is a widely used agent. In terms of disease control, the patient remains asymptomatic and with no evidence of disease eight months after discontinuing T-DM1.

## Conclusions

Portosinusoidal vascular disorder is a rare complication associated with treatment with T-DM1, and its diagnosis requires high clinical suspicion. Thrombocytopenia and alterations in liver enzymes are common side effects of T-DM1, but their presence should raise suspicion for PSVD. The only available treatment for T-DM1-induced PSVD is suspension of T-DM1.
